# Correlation between serum vitamin D level and neonatal indirect hyperbilirubinemia

**DOI:** 10.1186/s12887-018-1140-9

**Published:** 2018-05-26

**Authors:** Shahrokh Mehrpisheh, Azadeh Memarian, Abolfazl Mahyar, Negin Sadat Valiahdi

**Affiliations:** 1 Department of Neonatology, Mazandaran University of Medical Sciences, Mazandaran, Iran; 2 Department of Forensic Medicine, Iran University of Medical Sciences, Tehran, Iran; 3 Department of Pediatrics, Qazvin University of Medical Sciences, Tehran, Iran

**Keywords:** Infant, Vitamin D, Hyperbilirubinemia, Neonatal

## Abstract

**Background:**

Considering the significant prevalence of Neonatal Indirect Hyperbilirubinemia (NIH) and its irreversible neurological complications, identifying the factors involved in the prevalence of neonatal jaundice is essential. The present study was conducted to determine the relationship between serum vitamin D levels and the prevalence of NIH in infants admitted to Qods Hospital of Qazvin in Iran in 2015–16.

**Methods:**

In this case-control study, 30 term infants with NIH (the case group) were compared with 30 healthy, non- icteric, term infants (the control group) in terms of serum levels of 25-hydroxyvitamin D. The results were analyzed and compared between the two groups using t-test and the Chi-square test.

**Results:**

The mean and standard deviation of serum 25-hydroxyvitamin D levels were 10.76 ± 8.6 ng/dl in the case group and 14.88 ± 11.38 ng/dl in the control group. There were no significant differences between the two groups (*P* = 0.11).

**Conclusion:**

The results suggest the lack of a relationship between vitamin D levels and NIH. However, further prospective studies are needed to conclude that vitamin D has no role in the pathogenesis of NIH.

## Background

Neonatal indirect hyperbilirubinemia (NIH) is a prevalent issue among newborns [1]. NIH may has some detrimental complications such as long term neurologic deficits and death [2]. Any problem which rises bilirubin production and decreases conjugation can cause NIH [3]. Some of the causes of neonatal jaundice include blood group incompatibility, sequestration, G6PD deficiency, polycythemia and infections, while in most cases, there are no known causes [4]. Recent studies have shown the presence of vitamin D receptors in some of the cells derived from different tissues such as the liver, pancreas, brain and prostate as well as on the surface of immune cells, including lymphocytes and macrophages [5, 6]. Moreover, vitamin D activation occurs through 25-hydroxylation in the liver followed by 1-hydroxylation in the kidney [7, 8]. This metabolite can also be synthesized in various cells, including monocytes, skin cells and the placenta during pregnancy [9]. The liver tissue is not only involved in vitamin D synthesis, but also plays a key role in converting indirect bilirubin to direct bilirubin [10]. The metabolisms of bilirubin and vitamin D happen in two separate paths, but they may affect each other since one stage of their synthesis takes place in liver. The 25-hydroxylation stage, one of the main phases of vitamin D synthesis, takes place in the liver, as well as bilirubin conjugation [11]. So far, few studies have examined the relationship between hyperbilirubinemia and neonatal serum vitamin D [12, 13]. Given the high prevalence of jaundice and the importance of identifying its risk factors, understanding the relationship between these two can play a positive role in the diagnosis and treatment of neonatal jaundice. The present study was therefore conducted to investigate the relationship between serum vitamin D levels and hyperbilirubinemia in infants admitted to Qods Hospital in 2015–16.

## Methods

In this case-control study, 30 eligible infants with hyperbilirubinemia were compared with 30 healthy infants admitted to Qods Hospital in Qazvin, Iran, after obtaining a written consent from their parents. Hyperbilirubinemia was determined by bilirubin test that determine the bilirubin level in blood. Increasing of the bilirubin level more than 5 mg/dl was considered as hyperbilirubinemia. The infants were examined in terms of serum 25-hydroxyvitamin D level over 1 year. The term infants with a gestational age of 37–42 weeks and a postnatal age of three to 10 days, weighing 2500–4000 g and with no evidence of apparent anomalies, congenital anomalies, hematoma or symptoms suggesting infections as confirmed by a physician entered the study. Moreover, the infants with Rh or ABO incompatibility and with urinary infection, hypothyroidism, and children with direct or conjugated bilirubin> 0.8 were excluded from the study. The two groups were homogenized in terms of confounding and underlying variables such as age, gender, birth weight, gestational age, type of delivery, type of nutrition, place of residence, vitamin D supplementation during pregnancy, the mothers’ disease history, the mothers’ medication history, the families’ socioeconomic status, the mothers’ and the infants’ 25-hydroxyvitamin D level and the infants’ Ca (calcium), P (phosphorus), ALP (Alkaline phosphatas) and Mg (magnesium) levels. Infants whose mothers had a history of liver, kidney, thyroid and metabolic diseases such as diabetes or consumed a specific medication such as anticonvulsants were excluded from the study. The hospitalized icteric infants who were candidates for phototherapy based on the American Academy of Pediatrics entered the study after undergoing the required tests and the rejection of hemolytic icterus, sequestration, polycythemia and infections by a neonatologist. The infants’ weight, height and head circumference were measured by standard methods (the infants were weighed with a Seca weighing scale with a precision of 500 g). To measure serum 25-hydroxyvitamin D levels, 3 cm^3^ of blood was taken from the infants, its serum was separated, and stored in a freezer at − 20 °C until the test time. Considering the potential impact of phototherapy on serum vitamin D levels, the first serum sample of the icteric infants before the phototherapy was used for the tests. The 25-hydroxyvitamin D test was performed with the ELISA method (EIA or enzyme immunoassay), an ELISA Reader device called Awareness (made in the US), kits with a 2.7 ng/ml sensitivity and a batch number = 34,408, 33,737 (made by IDS in Germany) at the laboratory of Qods Hospital. All the biochemical and bilirubin tests were performed using photometry with an Auto Analyzer (Prestige 24i model, made in Japan), and the Ca level was measured with the help of Cresolphthalein Complexone, P with a UV test, ALP using the DGKC’s (German Society for Clinical Chemistry’s) method, Mg with Xylidyl Blue and total bilirubin with 2,4-dichloroaniline DCA. Based on the available sources of data, serum 25-hydroxyvitamin D levels were categorized as follows [14]: Very severe vitamin D deficiency = Less than 5 ng/ml, Severe vitamin D deficiency = 5–10 ng/ml, Vitamin D deficiency = 10–20 ng/ml, Suboptimal vitamin D provision = 20–30 ng/ml, Optimal vitamin D level = 30–50 ng/ml Upper normal = 50–70 ng/ml.

Data were statistically analyzed in SPSS-16. The frequency percentage, mean and standard deviation were used for the descriptive analysis of the data. The t-test was used to compare the two groups in terms of the mean quantitative variables and the Chi-squared test to compare them in terms of the mean qualitative variables. Data was statistically significant at *P* < 0.05.

## Results

**Fig. 1:**
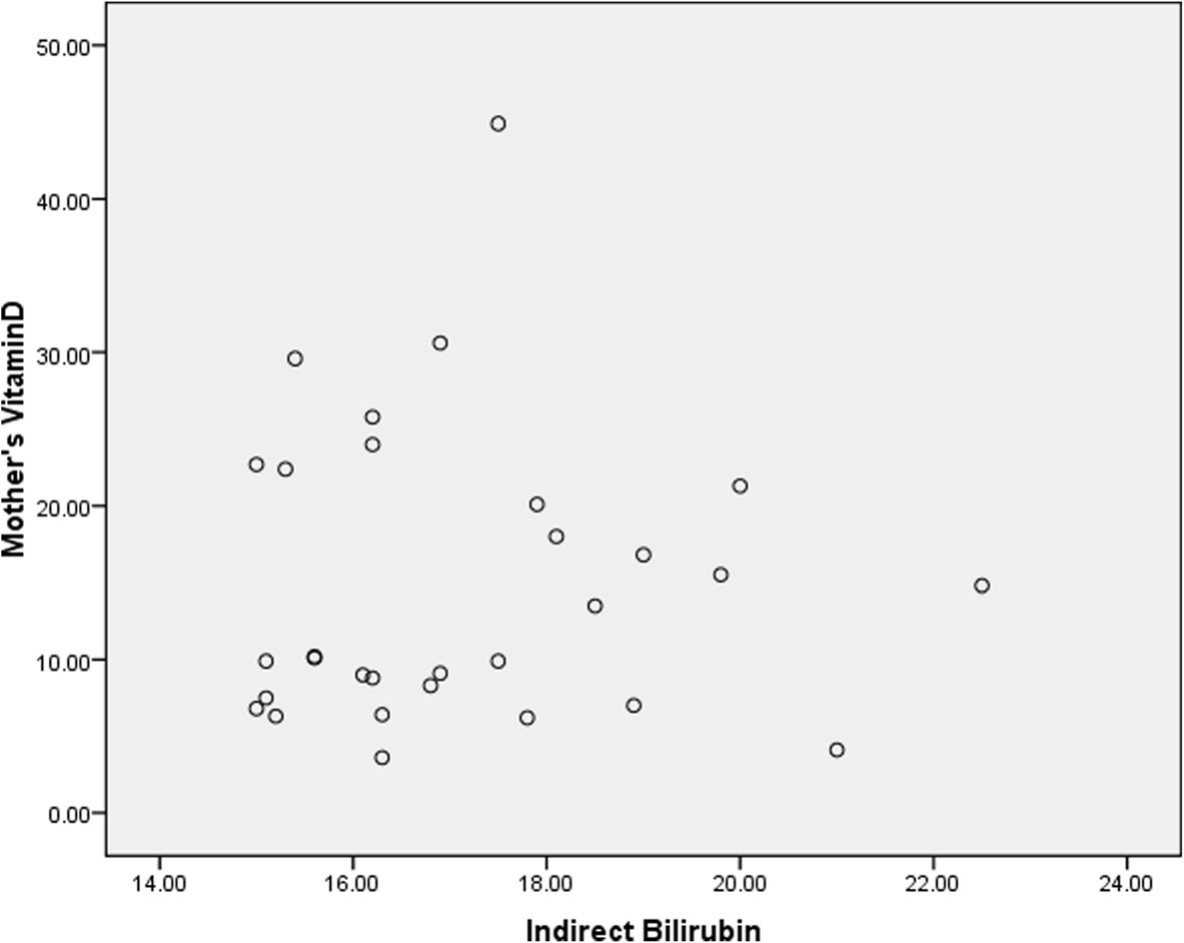
The relationship between serum vitamin D levels in the mothers and the infants’ indirect bilirubin levels: Indicates the lack of a relationship and correlation between the two variables (Pearson’s Correlation Coefficient = 0.97)

**Fig. 2:**
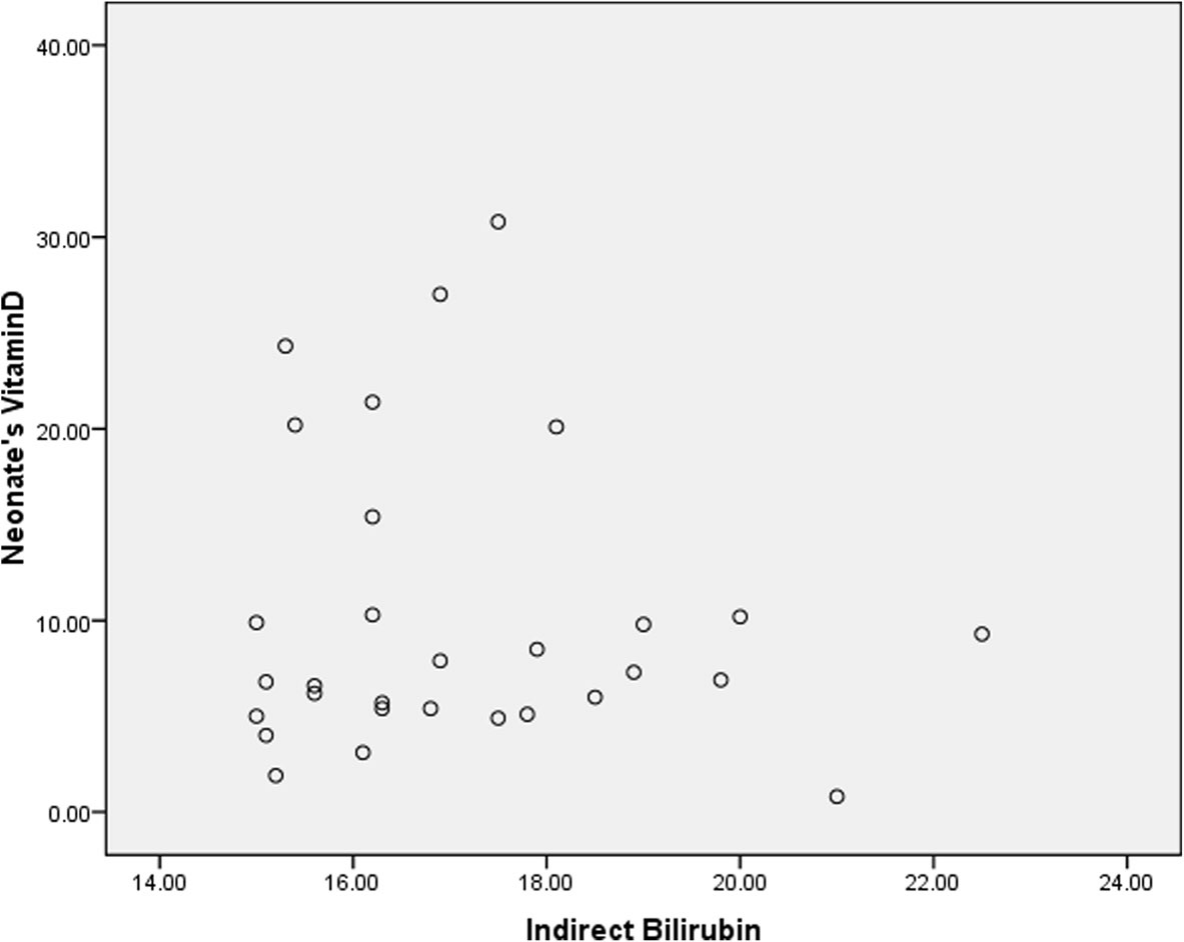
The relationship between serum vitamin D levels and indirect bilirubin in the infants: Indicates the lack of a relationship and correlation between the two variables (Pearson’s Correlation Coefficient = 0.64)

**Fig. 3:**
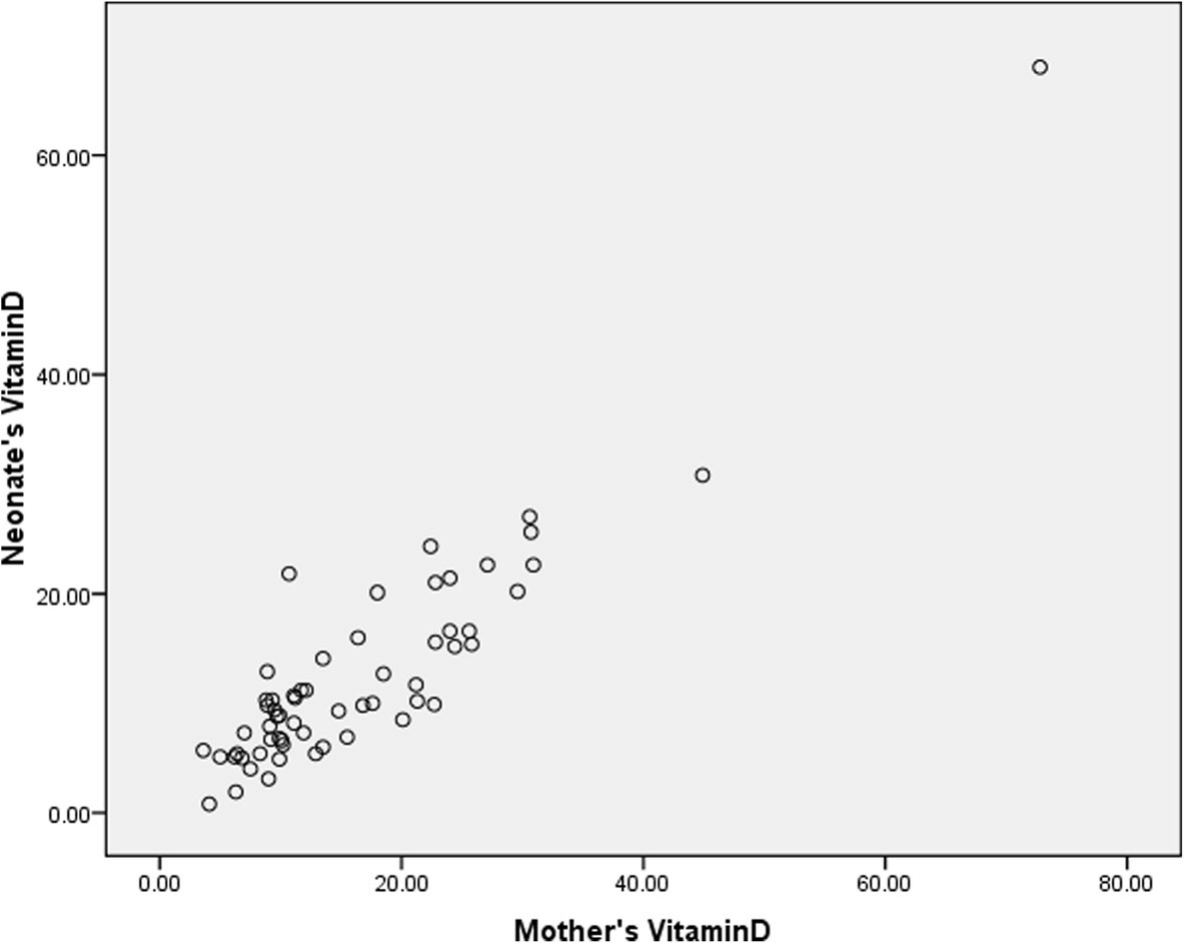
The relationship between serum vitamin D levels in the infants and the mothers: Indicates the lack of a relationship and correlation between the two variables (Pearson’s Correlation Coefficient = 0.00)

In this study, 60 term infants admitted to Qods Hospital of Qazvin were examined in two groups, including 30 infants with hyperbilirubinemia who were candidates for phototherapy in the case group and 30 healthy infants without jaundice in the control group. None of the subjects withdrew from the study, and the number of patients in each of the case and control groups remained the same until the very end. All the infants were breastfed and none had started receiving supplementation with formula. The mothers’ type of clothing was similar in the two groups. All the infants were cared for by their mothers and they were all Iranian in ethnicity. The infants were compared between the two groups in terms of demographic variables, including gender, age, birth weight, weight at the time of the visit (postnatal weight), height, head circumference, gestational age and type of delivery. No significant differences were observed between the two groups (Table 1); (*P* > 0.05). There were no significant differences between the two groups in terms of maternal variables such as the mothers’ age, BMI, vitamin D supplementation during pregnancy, number of deliveries and number of abortions (Table 2); (*P* > 0.05). There were no statistically significant differences between the two groups in terms of socioeconomic status, including the number of children, household size, the bedroom/household size ratio, family income, place of residence (urban/rural), the mother’s occupation and education and the father’s occupation and education (P > 0.05). The icteric infants had a mean hemoglobin of 16.63 ± 1.61, a mean hematocrit of 48.05 ± 4.8, a mean total bilirubin of 17.55 ± 2, and a mean indirect bilirubin of 17.12 ± 1.92. In the case group, 22 infants (73.3%) were hospitalized at Qods Hospital for phototherapy for 2 days, six infants (20%) for 3 days, one infant (3.3%) for 4 days and one infant (3.3%) for 5 days. The signs of jaundice appeared in the infants in the case group three to 7 days after birth and the time of blood sampling for measuring the vitamin D level was three to ten days after birth in both groups and the two groups were thus similar in terms of the time of taking the blood samples. There were no significant relationships between the two groups in terms of laboratory parameters such as Ca, P, Mg and ALP (Table 3); (*P* > 0.05). The mean serum vitamin D level in the mothers was 14.72 ± 9.60 in the case group and 17.71 ± 12.66 in the control group, suggesting the lack of a statistically significant relationship (Fig. 1); (*P* = 0.119). Finally, the two groups were compared in terms of serum vitamin D level in the infants (i.e. the main objective of the study). The mean serum vitamin D level was 10.76 ± 8.60 in the icteric infants (the cases) and 14.88 ± 11.38 in the healthy infants. Although the mean serum vitamin D level was lower in the infants with indirect hyperbilirubinemia than in the healthy infants, no significant differences were observed between the two groups and serum vitamin D level was not significantly related to neonatal jaundice (Figs. 2 and 3); (*P* = 0.119).

**Table 1:** A comparison of the mean demographic variables between the infants in the case and control groups and their relationships

Variables	Study group (*n* = 30)	Control group (n = 30)	*P*
Male/Female (n)	17/13	15/15	0.605
Postnatal age (day)	5.6 ± 1.75 (3–10)	6.26 ± 1.87 (3–10)	0.160
Birth weight (gr)	3266 ± 365 (2700–3950)	3287 ± 326 (2850–3820)	0.815
Postnatal weight (gr)	3215 ± 378 (2500–4100)	3244 ± 330 (2800–3800)	0.753
Head circumference (cm)	34.9 ± 1.01 (33–37)	34.98 ± 1.06 (33–37)	0.757
Height (cm)	50.43 ± 1.75 (46–53)	50.00 ± 1.41 (47–52)	0.297
Gestational age (week)	38.2 ± 1.3 (37–40)	38.4 ± 0.7 (37–40)	0.910
Delivey type: SVD/ C/S (n)	19/11	16/14	0.432
Breast feeding [n (%)]	30 (100)	30 (100)	0.321

SVD: Spontaneous vaginal delivery; C/S: Cesarean section

T-test and frequency and percentage are the tests used in this table

**Table 2:** Comparison of the mean demographic variables between the mothers in the case and control groups and their relationships

Demographic characteristics of the mothers’ cases and controls [mean ± SD (min-max)]			
	Study group (*n* = 30)	Control group (*n* = 30)	*P*
Mother age	27.13 ± 4.45 (17–33)	26.63 ± 6.85 (15–42)	0.739
Mother BMI	27.63 ± 4.41 (21.9–36.7)	25.41 ± 3.04 (21.5–33.6)	0.280
Abortion’s number	11	8	0.584
Delivery number	60	66	0.665
Mothers’ vitamin D use:			
Regularly [n (%)]	4 (13)	3 (10)	0.247
Irregularly [n (%)]	3 (10)	8 (27)	
None [n (%)]	23 (77)	19 (63)	

T-test and frequency and percentage are the tests used in this table

BMI: Body mass index

**Table 3:** A comparison of the mean laboratory parameters in the case and control groups and their relationships [mean ± SD (min-max)]

Variables	Study group (*n* = 30)	Control group (*n* = 30)	*P*
Ca (mg/dL)	9.21 ± 0.73 (7.7–10.7)	9.30 ± 0.58 (8.2–10.4)	0.256
P (mg/dL)	5.98 ± 0.82 (4.6–7.6)	5.73 ± 0.69 (4.3–7.3)	0.173
ALP (U/L)	409 ± 102 (247–647)	373 ± 106 (232–680)	0.895
Mg (mg/dL)	1.95 ± 0.20 (1.7–2.4)	1.99 ± 0.16 (1.7–2.3)	0.075
Neonate’s 25-OH vit D (ng/mL)	10.76 ± 8.60 (0.8–30.8)	14.88 ± 11.38 (5.1–68)	0.119
< 5 ng/mL [n (%)]	5 (17)	0 (0)	
5–10 ng/mL [n (%)]	16 (53)	10 (33)	
10–20 ng/mL [n (%)]	3 (10)	14 (47)	
20–30 ng/mL [n (%)]	5 (17)	5 (17)	
30–50 ng/mL [n (%)]	1 (3)	0 (0)	
50–70 ng/mL [n (%)]	0 (0)	1 (3)	
Mother’s 25-OH vit D (ng/mL)	14.72 ± 9.6 (3.6–44.9)	17.71 ± 12.66 (5.0–72.8)	0.307
< 5 ng/mL [n (%)]	2 (7)	0 (0)	
5–10 ng/mL [n (%)]	12 (40)	8 (27)	
10–20 ng/mL [n (%)]	7 (23)	12 (40)	
20–30 ng/mL [n (%)]	7 (23)	7 (23)	
30–50 ng/mL [n (%)]	2 (7)	2 (7)	
50–70 ng/mL [n (%)]	0 (0)	1 (3)	

*Ca* calcium, *P* phosphate, *ALP* alkaline phosphatase, *Mg* magnesium, *25-OH vit D* 25-hydroxy vitamin D

t-test and frequency and percentage tests are used in this table

## Discussion

The study found no relationships between serum vitamin D levels in infants and the prevalence of Neonatal Indirect Hyperbilirubinemia (NIH). Although the mean serum vitamin D level was lower in the infants with NIH compared to the healthy infants, no significant differences were observed between the two groups and serum vitamin D level was not significantly related to the prevalence of NIH [15]. About two-thirds of newborns develop clinical neonatal jaundice (serum bilirubin level > 5 mg/dl) and more than 97% of term and preterm infants develop biochemical hyperbilirubinemia (serum bilirubin level > 1 mg/dl); [16, 17]. Clinical guidelines recommend identifying the causes of hyperbilirubinemia and adopting effective prevention strategies [18]. Identifying the treatable etiology of hyperbilirubinemia and preventing its prevalence, which comprised the objectives of the present study, are essential. A similar prospective study by M. Mutlu et al. [19] investigated the relationship between vitamin D level and neonatal hyperbilirubinemia in term infants and compared vitamin D levels in infants with pathologic hyperbilirubinemia and healthy infants with normal or physiological levels of bilirubin. The results showed statistically significant differences between the control and case groups in terms of 25-hydroxyvitamin D levels (*P* = 0.01). A significant negative relationship was also observed between vitamin D levels and the parathyroid hormone in the infants (*P* = 0.03). Mutlu’s study was conducted over 1 year on infants aged three to ten days and born at a gestational age of 37–40 weeks who had a serum bilirubin level requiring phototherapy (group 1 or the case group) and healthy infants of the same age but without jaundice or with physiological jaundice only (group 2 or the control group). Both groups were examined at identical time periods in terms of their birth weight, gestational age, neonatal age, weight at the visit, type of delivery, gender, type of nutrition, mother’s age, mother’s type of clothing, place of residence (geographical region), vitamin D supplementation during pregnancy, the mother’s disease history and the mother’s medication history during the pregnancy, which could affect the level of vitamin D. Infants born to mothers with symptoms of chronic liver disease and kidney disease or those who regularly used anticonvulsants were excluded from the study [19]. Just as in the discussed study, the present study considered all these criteria, with few differences, including examining infants with a gestational age of 37 to 42 weeks and following up on all the infants in the control group until 15 days and eliminating the cases of physiological jaundice from the control group, such that even suspicions about very mild jaundice only in the sclera meant exclusion from the study. The groups were also homogenized in terms of their demographic variables and socioeconomic status. A study by E. Dan-Ierodiaconou et al. (1980) on the effect of phototherapy on vitamin D metabolism examined ten infants with jaundice under phototherapy treatment. The infants’ 25-OHD, 24, 25(OH)2D, Ca and P levels were measured before phototherapy and 24 and 48 h after the procedure was over. The mean weight of the infants was 3.4 kg, their mean age 64 h, their mean bilirubin level was 17.1 mg and their mean duration of phototherapy 83 h. The causes of jaundice included G6PD deficiency, Rh incompatibility, blood type incompatibility and unknown factors. For their phototherapy, the infants were placed under seven lamps positioned 60 cm above them and were fed with cow’s milk containing no vitamin D. The results of the study showed that the skin of infants with jaundice treated with phototherapy does not convert pro-vitamin D to active vitamin D [20–22]. A similar study by Gillies DR et al. (1984) on the effect of phototherapy in infants with neonatal jaundice on the production of vitamin D measured the 25(OH) VitD level before and 48 h after phototherapy. The results of the study showed no significant increase in active vitamin D levels 48 h after phototherapy [23]. Given the disparities in the results of the discussed studies and the potential impact of phototherapy on serum vitamin D levels, blood samples from the case group were sent to the laboratory for measuring the infants’ serum vitamin D level before starting phototherapy in the present study. In Mutlu’s study, hemogram, peripheral smear, reticulocyte count, blood group, direct Coombs, bilirubin, free T4, TSH, Ca, P, Mg, ALP, PTH and 25(OH) VitD testing was performed on all the infants. Ca, P, Mg, ALP, PTH, 25(OH) VitD and blood group testing was performed on all the mothers too. Given the existing imitations, the present study measured only Ca, P, Mg, ALP and 25(OH) VitD in the infants and 25(OH) VitD in the mothers. The objective of the present study was to evaluate the relationship between hyperbilirubinemia and serum vitamin D levels –not to assess the etiology of vitamin D deficiency; as a result, PTH, TSH and free T4 were not measured in this study. Considering the exclusion of infants with physiological icterus, the total and indirect bilirubin levels were not measured in the control group. Hemogram, peripheral smear, reticulocyte count, blood group, direct Coombs and G6PD activity testing were performed in all the icteric infants in the case group and the cases of hemolytic icterus, as diagnosed by the neonatologist, were excluded from the study. Mutlu’s research was a case-control study with a one-on-one design that was conducted on 51 infants, since some of the families that were entered into the study withdrew, and 30 infants with hyperbilirubinemia ultimately remained in group 1 and 21 healthy infants remained in group 2. In terms of laboratory parameters, bilirubin levels were significantly higher in group 1 compared to in group 2, while there were no statistically significant differences between the two groups in terms of Ca, P, Mg and ALP in the infants and Ca, P, Mg, ALP, PTH and 25(OH) VitD in the mothers. There was a significant difference between the two groups in terms of serum 25-hydroxy vitamin D levels and a significant negative relationship was also observed between vitamin D levels and PTH. Vitamin D deficiency was reported in 86% of the infants in group 1 and vitamin D inadequacy was reported in 7%. The case group had a significantly more severe degree of vitamin D deficiency compared to the controls, but there were no significant differences between the two groups in terms of vitamin D inadequacy. In the present study, none of the participants withdrew from the study because the parents were given adequate explanations, the physician visits were free and the families incurred no additional costs. The findings of this study showed a statistically significant difference between the control group and the infants with hyperbilirubinemia in terms of 25-hydroxyvitamin D level. There was also a significant negative relationship between vitamin D level and the parathyroid hormone in the infants. This disparity could be due to the high prevalence of vitamin deficiency D among Iranians, which led to an inadequate mean serum vitamin D level in both groups.

## Conclusion

The result of this study showed no relationships between vitamin D levels and NIH. In spite a fact that, a larger statistical population was examined in this study compared to previous studies, more extensive research or a cohort study or an animal study is still needed to generalize the result of this study.

### Limitations

A multivariable linear regression model with the infants’ indirect bilirubin as the outcome measure controlling for confounders and a regression model with infant Vitamin D as outcome and maternal Vitamin D as exposure to assess relationship would be helpful but unfortunately we weren’t able to do it in this study.

## Abbreviations

ALP: Alkaline Phosphatas
G6PD: Glucose-6-Phosphate Dehydrogenase
NIH: Neonatal Indirect Hyperbilirubinemia
PTH: Parathyroid hormone
